# Ultraviolet Photolysis of Chlorpyrifos: Developmental Neurotoxicity Modeled in PC12 Cells

**DOI:** 10.1289/ehp.11592

**Published:** 2008-09-09

**Authors:** Theodore A. Slotkin, Frederic J. Seidler, Changlong Wu, Emiko A. MacKillop, Karl G. Linden

**Affiliations:** 1 Department of Pharmacology and Cancer Biology, Duke University Medical Center, Durham, North Carolina, USA; 2 Department of Civil and Environmental Engineering, Duke University, Durham, North Carolina, USA; 3 Department of Civil, Environmental, and Architectural Engineering, University of Colorado at Boulder, Boulder, Colorado, USA

**Keywords:** chlorpyrifos, neurotoxicity, organophosphate insecticides, photolysis products, ultraviolet light

## Abstract

**Background:**

Ultraviolet photodegradation products from pesticides form both in the field and during water treatment.

**Objectives:**

We evaluated the photolytic breakdown of the organophosphate pesticide chlorpyrifos (CPF) in terms of both the chemical entities generated by low-pressure ultraviolet C irradiation and their potential as developmental neurotoxicants.

**Methods:**

We separated by-products using high-performance liquid chromatography and characterized them by gas chromatography/mass spectrometry. We assessed neurotoxicity in neuronotypic PC12 cells, both in the undifferentiated state and during differentiation.

**Results:**

Photodegradation of CPF in methanol solution generated CPF oxon and trichloropyridinol, products known to retain developmental neurotoxicant actions, as well as a series of related organophosphate and phosphorothionate derivatives. Exposure conditions that led to 50% degradation of CPF thus did not reduce developmental neurotoxicity. The degradation mixture inhibited DNA synthesis in undifferentiated cells to the same extent as native CPF. In differentiating cells, the products likewise retained the full ability to elicit shortfalls in cell number and corresponding effects on cell growth and neurite formation. When the exposure was prolonged to the point where 70% of the CPF was degraded, the adverse effects on PC12 cells were no longer evident; however, these conditions were sufficiently severe to generate toxic products from the methanol vehicle.

**Conclusions:**

Our results indicate that field conditions or remediation treatments that degrade a significant proportion of the CPF do not necessarily produce inactive products and, indeed, may elicit formation of even more toxic chemicals that are more water soluble and thus have greater field mobility than CPF itself.

The nearly ubiquitous exposure of the human population to organophosphate pesticides has raised increasing concern about their propensity to elicit developmental neurotoxicity at exposures that go undetected because of the absence of systemic signs of intoxication (Colborn 2006; Costa 2006; Landrigan 2001; Mileson et al. 1998; Slotkin 2005; Weiss et al. 2004). Chlorpyrifos (CPF), the most extensively studied of the organophosphates, disrupts neural cell replication and differentiation, axonogenesis, and synaptic function, culminating in behavioral deficits that have been noted both in animal models of developmental CPF treatment and in children in settings with high environmental CPF exposures (Rauh et al. 2006; Slotkin 1999, 2004, 2005). Although registration of CPF for use in the home has been withdrawn, it remains widely applied in agriculture, where issues remain about potential toxicity from runoff into natural water bodies and sources of drinking water or residues in food. In the field, CPF and other pesticides are exposed to conditions that lead to degradation, notably, photolysis from ultraviolet (UV) light. In water, these chemicals also can be degraded via environmental exposure to UV light and further transformed in water treatment processes. Recent studies of CPF photolytic products, CPF metabolites, and other organophosphates indicate that under these conditions, degradation involves the formation of a variety of known and unknown derivatives, some of which may retain developmental neurotoxicant features (Bavcon Kralj et al. 2007; Shemer and Linden 2006; Shemer et al. 2005).

In the present study, we evaluated the effects of different degrees of UVC irradiation of CPF and evaluated the potential neurotoxicity of the products in PC12 cells, a neurodevelopmental model derived from pheochromocytoma (Teng and Greene 1994) that recapitulates the major mechanisms and outcomes of CPF effects on the developing brain *in vivo* (Bagchi et al. 1995, 1996; Crumpton et al. 2000a, 2000b; Das and Barone 1999; Flaskos et al. 1994; Jameson et al. 2006b; Li and Casida 1998; Nagata et al. 1997; Qiao et al. 2001, 2005; Slotkin 1999, 2004, 2005; Song et al. 1998; Tuler et al. 1989; Yanai et al. 2002). PC12 cells enable detection of adverse effects on the cell cycle, an important target for CPF and other organophosphates (Slotkin 1999, 2004, 2005), whereas primary neurons do not maintain their mitotic ability in culture and differentiate in a heterogeneous fashion. PC12 cells undergo coordinated differentiation into distinct neuronal phenotypes when nerve growth factor (NGF) is added, exiting the mitotic cycle and growing neuritic projections (Fujita et al. 1989; Song et al. 1998; Teng and Greene 1994). In the present study, we examined the effects of CPF before and after photolysis on both undifferentiated and differentiating PC12 cells with regard to DNA synthesis and indices of cell number and neurite outgrowth. Unlike hepatocytes or myocytes, each neural cell contains a single nucleus, so measuring DNA content evaluates the number of cells (Winick and Noble 1965). We also assessed total protein, which increases with cell growth, and membrane protein, which rises with the formation of neurites (Abreu-Villaça et al. 2005; Jameson et al. 2006a; Slotkin et al. 2007b; Song et al. 1998).

## Materials and Methods

### Photolysis

We dissolved CPF (purity > 99%; Chem Service, West Chester, PA) in methanol (VWR Scientific, West Chester, PA) and carried out photodegradation experiments in a collimated-beam low-pressure UV bench reactor, configured with four mercury vapor germicidal lamps (ozone-free, General Electric no. G15T8), emitting monochromatic light at 254 nm. We placed the test solution in a 70 × 50 mm crystallization dish, which was sealed with a quartz cover to prevent evaporation, and exposed it to UVC irradiation. Temperature in the dish was maintained at 24°C by a coiled water cooling system, and the solution was mixed on a stir plate to ensure complete mixing and consistent batch conditions. We determined incident irradiance using a calibrated radiometer (IL1700, SED 240/W; International Light, Peabody, MA) and calculated delivered UVC fluence with a spreadsheet program that included lamp spectrum, solution absorbance, exposure time, and incident irradiance (Bolton and Linden 2003). At specific delivered UVC fluence intervals, about 10 mL of sample was collected with a syringe and sealed in a borosilicate vial for the biological determinations. We determined the final CPF concentrations by high-performance liquid chromatography (HPLC) and conducted control experiments with pure methanol vehicle under the same irradiation conditions.

### Chemical analysis

We measured concentrations of CPF during photolysis with a Varian Pro Star HPLC (Varian, Inc., Palo Alto, CA) equipped with a polychromatic diode array detector and a 4.6 × 150 mm C18 reverse-phase column (Alltech Associates, Deerfield, IL). We used isocratic elution with a mobile phase of acetonitrile and water (80/20 vol/vol) at a flow rate of 1 mL/min. Under these conditions, the retention time for CPF was 7.9 min. For by-product identification, we used a Shimadzu GC/MS-QP2010 gas chromatograph/mass spectrometer (GC/MS) equipped with a 15-m RTX-5MS column (film thickness, 0.25 μm; i.d., 0.25 mm).

### Cell cultures

Because of the clonal instability of the PC12 cell line (Fujita et al. 1989), we performed the experiments on cells that had undergone fewer than five passages and repeated all studies several times with different batches of cells. As described previously (Crumpton et al. 2000a; Qiao et al. 2003; Song et al. 1998), we seeded 3 × 10^6^ PC12 cells (American Type Culture Collection 1721-CRL; obtained from the Duke Comprehensive Cancer Center, Durham, NC) onto poly-d-lysine–coated plates in RPMI-1640 medium (Invitrogen, Carlsbad, CA) supplemented with 10% inactivated horse serum (Sigma Chemical Co., St. Louis, MO), 5% fetal bovine serum (Sigma), and 50 μg/mL penicillin streptomycin (Invitrogen). Cells were then incubated with 7.5% CO_2_ at 37°C and the medium was changed every 2 days. For studies in the undifferentiated state, the medium was changed 24 hr after seeding to include CPF or CPF photolysis products. Because of its poor water solubility, CPF was dissolved in methanol, the same medium used for the photolysis exposures. The final concentration of methanol in the culture medium ranged from 16 to 47 mM (0.05–0.15%), and the same vehicle was added to the control cultures. By itself, methanol had no effect on proliferation, growth, or differentiation of PC12 cells, as established previously (Das and Barone 1999) and as confirmed in preliminary studies for these experiments. In our previous work on organophosphates in the PC12 model, we typically used dimethylsulfoxide (DMSO) vehicle (Qiao et al. 2001, 2003; Song et al. 1998), but we found that photolysis of DMSO produced neurotoxic products.

For studies in differentiating cells, we seeded 3 × 10^6^ cells and 24 hr later changed the medium to include 50 ng/mL of 2.5S murine NGF (Invitrogen); we examined each culture under a microscope to verify the subsequent outgrowth of neurites. CPF or CPF photolysis products were added concurrently with the start of NGF treatment, and cultures were maintained for 6 days, with the test agents included with every medium change.

We chose the CPF concentrations on the basis of previous work. Given our objective to evaluate the loss of activity upon photolysis, our strategy was to elicit both threshold and robust responses for each of the effects evaluated. Accordingly, we used 10 or 30 μM CPF in undifferentiated cells, concentrations that span the range from low to high effects on DNA synthesis, and 30 μM CPF for studies in differentiating cells (Bagchi et al. 1995; Crumpton et al. 2000b; Das and Barone 1999; Jameson et al. 2006b; Qiao et al. 2001, 2003, 2005; Slotkin et al. 2007b; Song et al. 1998).

### DNA synthesis

We introduced CPF or CPF photolysis products for 1 hr in undifferentiated cells, and then, to initiate the measurement of DNA synthesis, we changed the medium to include 1 μCi/mL [^3^H]thymidine (specific activity, 2 Ci/mmol; GE Healthcare, Piscataway, NJ) along with the continued inclusion of the test substances. After 1 hr, the medium was aspirated and cells were harvested in ice-cold water. Duplicate aliquots of each sample were treated with 10% trichloroacetic acid and sedimented at 1,000 × *g* for 15 min to precipitate macro molecules. The resulting pellet was washed once with additional trichloro acetic acid and then with 75% ethanol. The final pellet was hydrolyzed with 1 M KOH overnight at 37°C and neutralized with 6 M HCl; the DNA was then precipitated with ice-cold 5% trichloroacetic acid and resedimented. The supernatant solution, comprising solubilized RNA and protein, was discarded. The DNA-containing pellet was hydrolyzed in 5% trichloroacetic acid for 15 min at 90°C and resedimented, and an aliquot of the supernatant solution was counted for radiolabel. We assayed another aliquot for DNA spectrophotometrically by absorbance at 260 nm. Previous work has demonstrated quantitative recovery of DNA by these techniques (Bell et al. 1986; Slotkin et al. 1984). Incorporation values were corrected to the amount of DNA present in each culture to provide an index of DNA synthesis per cell (Winick and Noble 1965).

### DNA and protein content

We evaluated DNA content, total protein, and membrane protein after 6 days of continuous exposure to NGF and either CPF or CPF photolysis products. The medium was aspirated and the culture was rinsed with a buffer consisting of 154 mM NaCl and 10 mM sodium phosphate (pH 7.4). Cells were harvested in ice-cold buffer and homogenized (Polytron, Brinkmann Instruments, Westbury, NY), and aliquots were withdrawn for measurements of DNA and protein using dye-binding methods (Smith et al. 1985; Trauth et al. 2000). To prepare the cell membrane fraction, we sedimented another aliquot of the homogenate at 40,000 × *g* for 10 min; and the pellet was then washed and resedimented. The membrane pellets were then resuspended and analyzed for protein. We selected representative samples for each treatment, examined them with light microscopy, and counted cells to verify that reduced DNA content connoted fewer cells.

### Data analysis

We conducted CPF photolysis in three separate sets of samples, with each sample prepared several weeks apart, and then tested them in multiple batches of PC12 cells. Results are reported as mean ± SE. Because of the multiple treatments in each experiment, significant differences were first established by a global analysis of variance (ANOVA) incorporating all treatments, followed by Fisher’s protected least significant difference to evaluate differences between specific treatment groups. The initial test included sample batch and cell batch; however, because the treatment effects were the same, we then combined the results across batches after normalization to account for the differing absolute values for each batch of cells. Significance was assumed at *p*< 0.05.

## Results

### Photolysis

**Figure 1:**
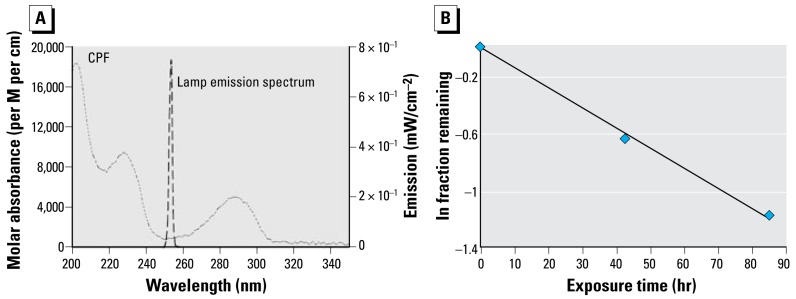
(*A*) Molar absorption coefficient of CPF across UV wavelengths, and emission spectrum for the UV lamp. (*B*) Degradation of CPF by direct photolysis at 254 nm, shown as natural log of fraction remaining, demonstrating a first-order decline with time; we obtained the same result plotting the results against fluence (data not shown).

Direct photolysis of CPF can be described as a pseudo-first-order reaction following these two equations (Schwarzenbach et al. 2003)


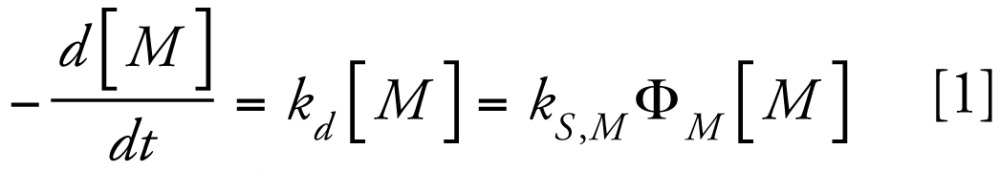



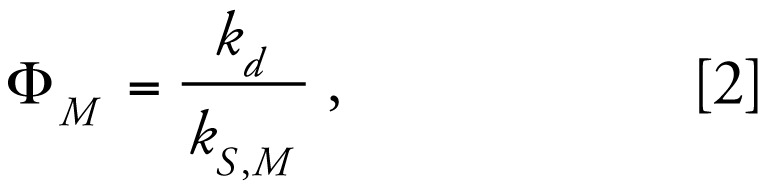


where


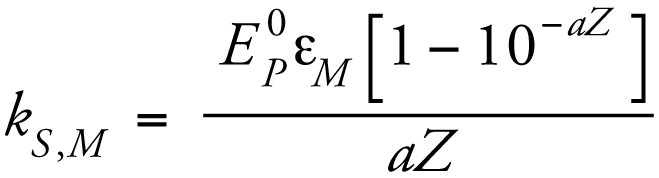


is the specific rate of light absorption (*E* mol^−1^ sec^−1^); Φ_M_ is the quantum yield; *k_d_* is the pseudo-first-order rate constant; [M] is the concentration of target compound; *E_P_*^0^ is incident photon irradiance (10^−3^ *E* cm^−2^ sec^−1^); ɛ*_M_* is molar absorbance (M^−1^ cm^−1^) of the target compound; *Z* is the solution depth; and *a* is the light absorbance by the solution. Using a spectro radiometer, we confirmed that the UVC lamp emitted monochromatically at 254 nm, with insignificant contributions from other wavelengths (Figure 1A). CPF had strong absorbance bands at 230 nm and 290 nm, but a local absorption minimum at 254 nm, indicating limited susceptibility to direct photodegradation by low-pressure UVC irradiation. Therefore, the direct photolysis of CPF in methanol progressed slowly because of the low absorbance, combined with the fact that CPF has a low quantum yield. The initial stock CPF concentration was 15.83 mM, and the final concentrations after short-term and long-term UVC treatments were 8.30 mM and 4.84 mM, respectively, corresponding to about 50% and 70% reduction of the initial CPF. Applying the photo degradation data to the integrated form of Equation 1 confirmed the adherence to the predicted pseudo-first-order reaction (Figure 1B).

The application of GC/MS made it possible to determine the major photodegradation by-products by comparing mass spectra of the unknowns with a National Institute of Standards and Technology mass spectral library search (NIST 2008) and through analysis of the mass fragmentation patterns (Table 1). The products included agents whose potential for developmental neurotoxicant actions have been characterized in the PC12 model, such as CPF oxon and trichloropyridinol(2-hydroxyl-3,5,6-trichloropyridine) (Qiao et al. 2001), as well as novel products that retain organophosphate or phosphorothionate characteristics. In comparing the products obtained in methanol solution to those obtained in water, most of the products were identical: *O*,*O*-diethyl-methyl thiophosphate, *O*,*O*-diethyl thiophosphate, 2-hydroxyl-3,5,6-trichloropyridine (trichloropyridinol), 3,6-dichloro-2-[pyridinyl-*O*,*O*-ethyl] thiophosphate, and CPF oxon. Whereas photolysis in methanol solution also produced dimethyl ethyl phosphate, water solution produced the similar (but not identical) product, *O*,*O*-diethyl phosphate. Photolysis in water also produced 3,5,6-trichloro-2-pyridinyl acetic acid, which was not found with the methanol solution, although this is obviously quite similar to trichloropyridinol. We found none of these products in untreated CPF solutions.

### Undifferentiated cells

**Figure 2:**
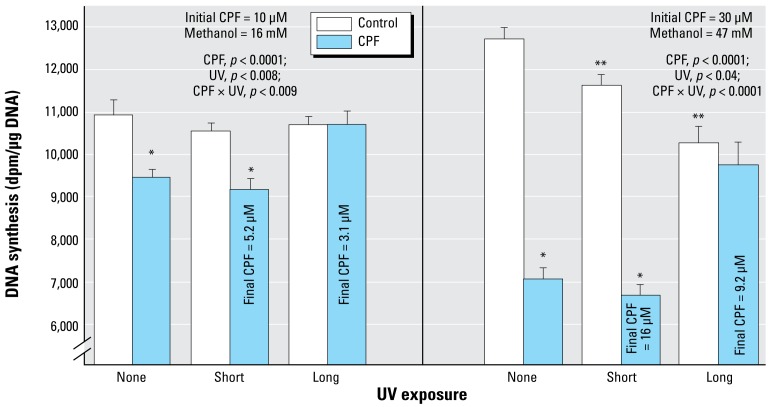
Effects of CPF and CPF photolysis products on DNA synthesis in undifferentiated PC12 cells (2-hr exposure, no serum; *n* = 8). ANOVA *p*-values across all treatments and UV exposures appear within the panel. For lower-order tests, *p*< 0.05 for *CPF compared with controls; and **UV-exposed control compared with control/none.

In agreement with earlier work (Qiao et al. 2001; Slotkin et al. 2007a, 2007b; Song et al. 1998), exposure of undifferentiated PC12 cells to 10 or 30 μM CPF produced concentration-dependent reduction in DNA synthesis, with the effect ranging from about 15% inhibition at the lower concentration (left) to nearly 50% inhibition at the higher concentration (right) (Figure 2). The shorter UVC exposure reduced the CPF concentration by about half, to 5.2 μM and 16 μM, respectively. Nevertheless, DNA synthesis was inhibited to the same extent as for the native compound. However, increasing the UVC exposure to the point where 70% of the CPF was lost effectively eliminated the ability to inhibit DNA synthesis. By itself, UVC exposure of the methanol vehicle also had a smaller, significant inhibitory effect that was revealed at the higher concentration, likely representing formation of toxic products such as formaldehyde and formic acid.

### Differentiating cells

**Figure 3:**
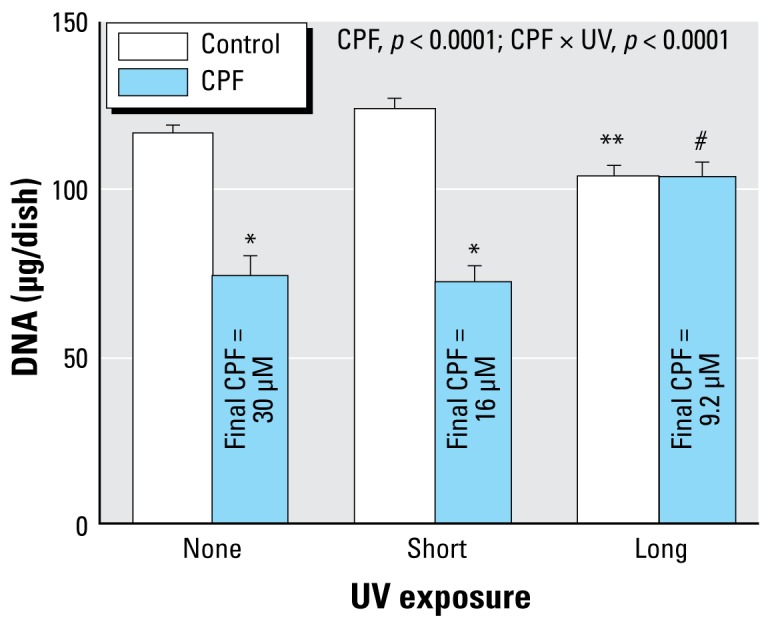
Effects of CPF and CPF photolysis products on DNA content in differentiating PC12 cells treated with the indicated agents along with NGF for a total of 6 days (47 mM methanol; *n* = 8). ANOVA *p*-values across all treatments and UV exposures appear within the panel. Lower order tests, *p* < 0.05 for *CPF compared with control; **UV-exposed control compared with control/none; and ^#^UV-exposed CPF compared with CPF.

**Figure 4:**
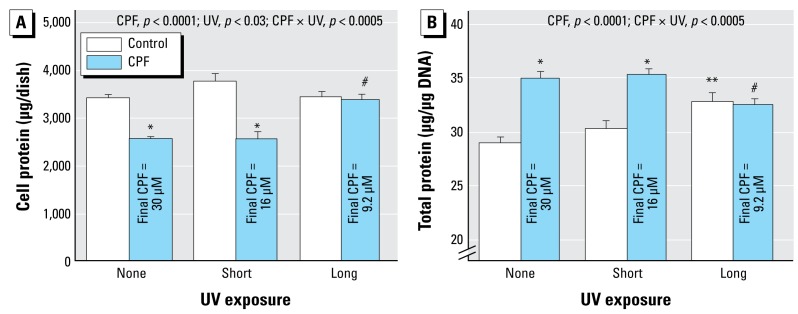
Effects of CPF and CPF photolysis products on total cell protein (*A*) and protein:DNA ratio (*B*) in differentiating PC12 cells treated with the indicated agents along with NGF for a total of 6 days (47 mM methanol; *n* = 8). ANOVA *p*-values across all treatments and UV exposures appear within the panel. Lower-order tests, *p* < 0.05 for *CPF compared with control; **UV-exposed control compared with control/none; and ^#^UV-exposed CPF compared with CPF.

When CPF was introduced simultaneously with NGF and differentiation allowed to proceed for 6 days, we observed a substantial reduction in the total number of cells as monitored by DNA content (Figure 3). Short-term UVC exposure, which reduced the effective CPF concentration by about half, failed to alter the deficit, but longer UVC exposure (70% destruction) completely eliminated the cell loss. Again, UVC irradiation of methanol had a minor but significant effect by itself. We obtained similar results for total cell protein: CPF elicited a significant decrease that was not prevented by the shorter UVC irradiation but was prevented by the longer UVC treatment (Figure 4A). However, the effects on total protein were significantly smaller than those on DNA content (CPF × measure, *p* < 0.0001; CPF × UV × measure, *p* < 0.002), reflecting a greater effect of CPF on cell number than on cell growth (Qiao et al. 2001; Slotkin et al. 2007a, 2007b; Song et al. 1998). Accordingly, we also evaluated the total protein:DNA ratio as an index of relative cell size. By itself, CPF evoked an increase in the ratio, and again, short UVC irradiation did not prevent the effect (Figure 4B); longer irradiation eliminated the effect of CPF, whereas the methanol vehicle once more showed a significant effect by itself.

**Figure 5:**
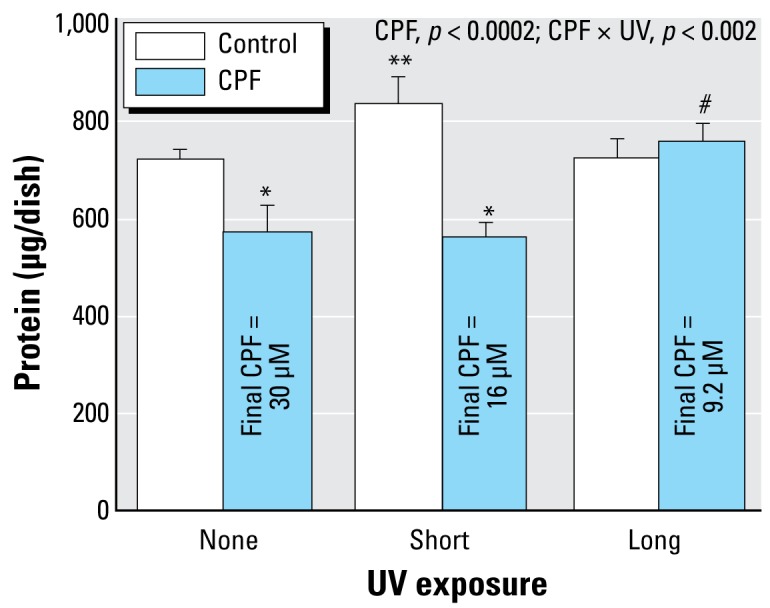
Effects of CPF and CPF photolysis products on membrane protein in differentiating PC12 cells. We treated cells with the indicated agents along with NGF for a total of 6 days (47 mM methanol; *n* = 8). ANOVA *p*-values across all treatments and UV exposures appear within the panel. Lower-order tests, *p* < 0.05 for *CPF compared with control; **UV-exposed control compared with control/none; ^#^UV-exposed CPF compared with CPF.

CPF exposure also reduced membrane protein (Figure 5) but to a lesser extent than the effect on DNA content. The effect was maintained or even exacerbated by short-term UVC exposure, whereas long-term exposure eliminated the deficit.

## Discussion

The main finding of these studies is that photodegradation of CPF does not necessarily lead to biological inactivation of adverse effects directed at developing neurons, one of the major concerns for CPF as well as other organophosphates (Colborn 2006; Costa 2006; Pope 1999; Slotkin 2005; Weiss et al. 2004). Although UV irradiation is a characteristic of both field conditions and engineered water purification, a number of features make CPF more problematic than several other organophosphates that have been evaluated under these conditions. CPF photodegradation products include the oxon and other bioactive molecules not necessarily shared by degradation of several other organophosphates (Bavcon Kralj et al. 2007). Although CPF is less water soluble and therefore less likely to run off than, for example, parathion, several of the degradation products identified here are far more soluble and thus may elicit effects on aquatic populations. For example, trichloropyridinol is the main CPF metabolite found in humans and other organisms and is known to impair neurite outgrowth (Howard et al. 2005), elicit gliotoxicity (Zurich et al. 2004), affect nuclear transcription factors involved in neurodifferentiation (Schuh et al. 2002), and inhibit mitotic activity in both neuronotypic and gliotypic cells (Qiao et al. 2001). In combination with CPF, trichloropyridinol increases the net impact on aquatic organisms (Caceres et al. 2007), emphasizing how photodegradation mixtures may actually show augmented adverse effects.

Although we have already characterized the ability of both CPF oxon and trichloropyridinol to act as developmental neurotoxicants (Qiao et al. 2001), it is likely that several other breakdown products are also biologically active. In particular, both CPF oxon and trichloropyridinol are less potent inhibitors of neuronal cell DNA synthesis than the parent compound (Qiao et al. 2001); because we found no loss of this biologic activity with up to 50% photodegradation, it is clear that the remaining products must have equivalent or greater effects than CPF itself. It will thus be critical to assess which molecules are the most active, the degree to which these appear in ecosystems, and the extent to which they accumulate in wildlife and humans. Our results also point to a clear line of demarcation between UVC exposures that degrade 50% and those that degrade 70% of the original CPF concentration, because biologic activity was lost with the more prolonged treatment paradigm. At these longer exposures, the degradation products were themselves likely broken down by photolysis, leading to inactive compounds. It is not clear, however, whether that scenario is relevant to typical UV exposure because this also produced sufficient degradation of the methanol to elicit toxic compounds. Again, future studies should address whether complete photodegradation is a reasonable outcome under real-life conditions.

There are a number of limitations in the interpretation of our results regarding UV photolysis as it occurs in water treatment or as a result of sunlight exposure in the field. First, we examined the effects of exposure to a fixed wavelength of 254 nm, rather than sunlight or other UV combinations. Notably, however, earlier work with a Suntest apparatus emitting in the range of 300–800 nm, as well as a variety of low-pressure UV light sources, found the same major breakdown products likely to possess biologic activity, including CPF oxon and trichloropyridinol (Barcelo et al. 1993; Racke 1993; Walia et al. 1988); accordingly, it is highly likely that the products found here are representative of the mixture after other types of photolytic exposures. Second, we had to use a vehicle that permits dissolution of CPF in high concentrations because of the need for subsequent dilution in culture medium. Either DMSO or methanol is suitable for use in PC12 cells because neither affects cell viability, replication, or differentiation in concentrations comparable with, or even higher than, those used here (Das and Barone 1999; Qiao et al. 2001, 2003; Song et al. 1998). Because the photolytic products of DMSO proved to be neurotoxic to PC12 cells, we restricted our studies to methanol vehicle; it is therefore important to note that the CPF breakdown products in methanol vehicle were virtually identical to those obtained in water, and included the major products identified with other sources as already described. A third issue is that, although we were able to identify the breakdown products based on spectral analysis, we could not quantify them because of the lack of standards for many of the compounds. In light of earlier studies, however, it is highly likely that CPF oxon and trichloropyridinol represent a large proportion of the total (Barcelo et al. 1993; Racke 1993; Walia et al. 1988), and as discussed above, there is no question about the role of these agents as contributors to developmental neurotoxicity. The fact that other products retain phosphorothionate and organophosphate characteristics suggests strongly that the other components in the photolytic mixture are also likely to participate in the net outcome.

The present findings indicate that photodegradation of CPF, and potentially other environmental toxicants, does not necessarily produce inactive products, and indeed, some of the resultant compounds and mixtures may actually exhibit equal or greater toxicity than the parent chemical. These factors should be taken into account in determining whether the disappearance of an agent in the field or during remediation treatments actually connotes a safer outcome.

## Figures and Tables

**Table 1:** Structure and mass spectra of photolysis by-products of CPF.

Name, RT	Proposed structure	Spectral data (*m*/*z*)
Dimethyl ethyl phosphate^a^ (RT = 5.28 min)	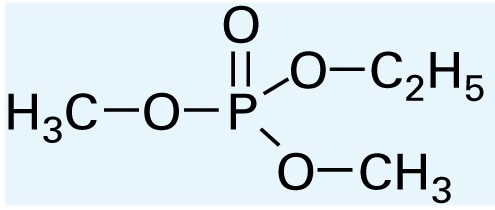	153 (M+), 127, 113, 109, 96
*O*,*O*-Diethyl phosphate^b^ (RT = 5.45 min)	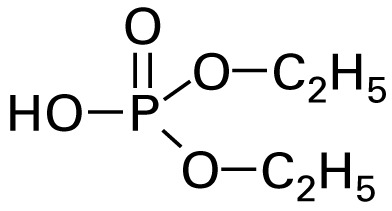	154 (M+), 127, 109, 95, 79
*O*,*O*-Diethyl-methyl thiophosphate (RT = 6.51 min)	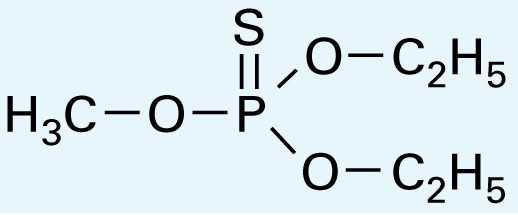	184 (M+), 156, 129, 107, 95, 79
*O*,*O*-Diethyl thiophosphate (RT = 6.97 min)	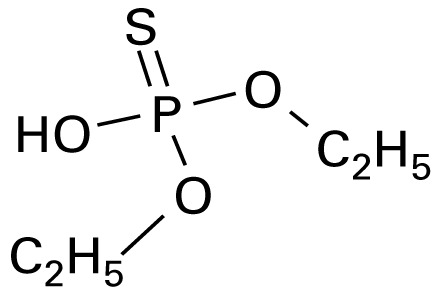	170 (M), 141, 113, 95, 81
2-Hydroxyl-3,5,6-trichloro pyridine (RT = 10.21 min)	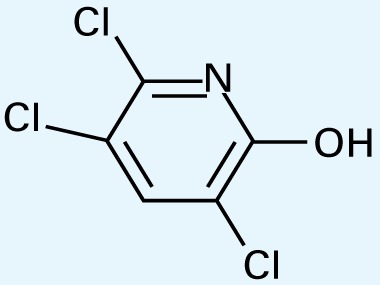	197 (M+), 169, 134, 107
3,5,6-Trichloro-2-pyridinyl acetic acid^a^ (RT = 10.27 min)	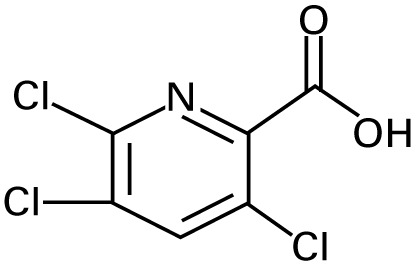	226 (M+), 210, 182, 146, 110
3,6-Dichloro-2-[pyridinyl-*O*,*O*-ethyl] thiophosphate (RT = 15.14 min)	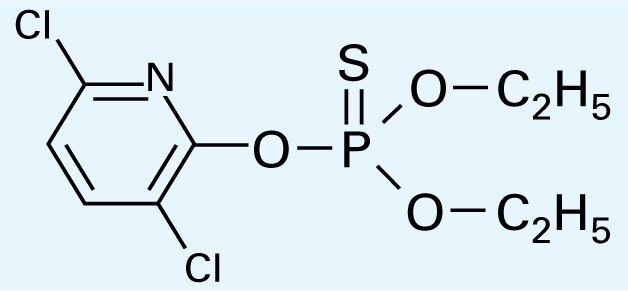	315 (M+), 280, 252, 224, 163, 97
CPF (RT = 17.7 min)	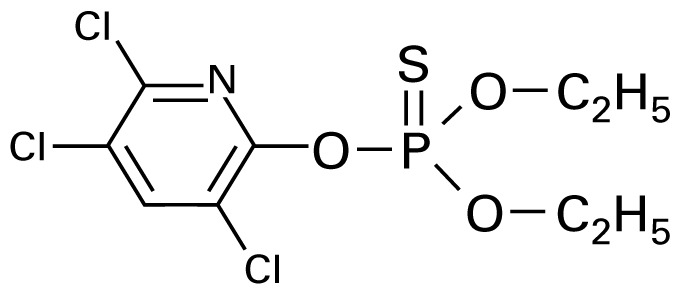	349 (M+), 314, 286, 258, 197, 125, 97
CPF oxon (RT = 20.88 min)	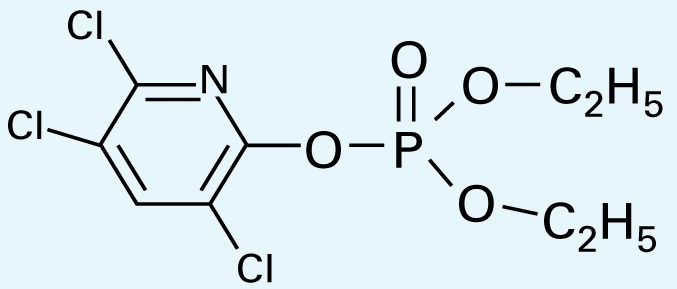	333 (M+), 310, 254, 203, 193

RT, retention time

a Found in methanol solution only.

b Found in water solution only.
